# Calcium Deficiency Decreases Bone Mass without Affecting Adiposity in Ovariectomized Rats Fed a High-Fat Diet

**DOI:** 10.3390/nu16040478

**Published:** 2024-02-07

**Authors:** Jay J. Cao, Brian R. Gregoire

**Affiliations:** USDA, Agricultural Research Service, Grand Forks Human Nutrition Research Center, Grand Forks, ND 58202, USA

**Keywords:** calcium, high-fat diet, bone, obesity, ovariectomy body composition

## Abstract

Obesity induced by a high-fat (HF) diet increases bone resorption and/or decreases bone formation, resulting in reduced bone mass and strength in various animal models. Studies showed that Ca intake is a modifiable factor for osteoporosis and obesity. This study investigated whether Ca deficiency affects bone structure and adiposity in ovariectomized (OVX) rats fed a HF diet. We hypothesized that Ca deficiency further decreases bone mass and increases fat mass in HF-fed OVX rats. Forty-seven OVX at 6-month-old were randomly assigned to four groups in a 2 × 2 factorial design: normal-fat (NF, 10% fat as energy) or HF (45% fat as energy) diet with either low Ca (LC, 1 g/4057 kcal) or normal Ca (NC, 6 g/4057 kcal). In addition, 12 sham-operated rats at 6 months old were fed a NFNC diet as a control for the OVX procedure. Rats were fed the respective diet for 4 months. Dietary Ca content did not affect body weight, fat mass, lean mass, food intake, energy intake, and serum cytokines. Compared to NC, LC resulted in lower tibial bone volume/total volume (BV/TV, *p* < 0.01), connectivity density (*p* < 0.01), trabecular number (Tb.N, *p* = 0.01), bone mineral density (BMD, *p* < 0.01), and femur weight (*p* < 0.01), femur content of Ca (*p* < 0.01), Cu (*p* = 0.03), Zn (*p* < 0.01), and greater trabecular separation (Tb.Sp, *p* < 0.01) at proximal tibia indicating bone structure deterioration. Compared to rats on the NF diet, animals fed the HF had lower BV/TV (*p* = 0.03) and Tb.N (*p* < 0.01) with greater body weight (*p* < 0.01), fat mass (*p* < 0.01), Tb.Sp (*p* = 0.01), the content of Ca, Cu, and Zn in the femur, and serum leptin (*p* < 0.01). There were no significant interactions between Ca and fat for body composition and bone structural parameters. Compared to Sham, OVX resulted in greater body weight and fat mass. The trabecular bone structure of the tibia, but not the cortical bone, was significantly impaired by the OVX procedure. These data indicate that inadequate Ca intake and a high-fat diet have independent negative effects on bone structure and that Ca deficiency does not affect adiposity in OVX rats.

## 1. Introduction

Bone metabolism is influenced and regulated by various dietary, physical, and hormonal factors. Among many dietary macro- and micronutrients affecting bone health and osteoporosis risk, dietary Ca is considered the most important nutrient, and its intake is likely to be inadequate [1]. Low Ca intake is considered one of the modifiable risk factors for osteoporosis [2]. A low Ca diet lowers serum concentrations of 25(OH)D_3_ and Ca^2+^ and impairs bone development by increasing serum concentrations of 1,25(OH)_2_D and parathyroid hormone in rats [3]. Low dietary Ca increases bone turnover by increasing the number of osteoclasts and osteoblasts on the trabecular bone surface and the number of progenitors of osteoclasts and osteoblasts in bone marrow [4].

With obesity and osteoporosis growing in prevalence, the impact of obesity on bone metabolism has been extensively investigated in various animal models and human studies [5,6]. Evidence supports that obesity is a risk factor for low bone mass and osteoporosis [5,7]. Obesity-induced by high-fat diets increases bone resorption and/or decreases bone formation, resulting in reduced bone mass and strength [8]. The mechanisms through which obesity or a high-fat diet (HF) affects bone metabolism have been extensively reviewed elsewhere [7].

Limited data suggest that dietary Ca intake may regulate energy metabolism and obesity risk [9,10]. For example, mice fed a low-Ca diet had accelerated weight gain and fat accretion, while a high-Ca diet inhibited lipogenesis and suppressed fat accretion and weight gain when animals maintained identical energy intakes [11]. A meta-analysis found a decrease in body mass index of 1.1 kg/m^2^ for every 800 mg/d increase in Ca intake in humans [12].

Whether Ca deficiency increases adiposity and exacerbates bone loss in HF diet-induced obesity in ovariectomized animals has not yet been investigated. Using ovariectomized (OVX) rats as a model, we hypothesized that Ca deficiency would increase adiposity and induce greater bone loss in rats fed a HF diet than those fed a normal-fat (NF) diet.

## 2. Materials and Methods

### 2.1. Animals, Diet, and Treatments

A total of 47 female OVX (to mimic the postmenopausal osteoporosis model) and 12 sham-operated (Sham) Sprague Dawley rats at 6 months old were purchased from Charles River Laboratories (Wilmington, MA, USA). Rats were shipped one week after the OVX operation. The study was conducted according to the guidelines of the Declaration of Helsinki, reviewed and approved by the United States Department of Agriculture, Agricultural Research Service, Grand Forks Human Nutrition Research Center Institutional Animal Care and Use Committee (GFHNRC-JC8, 6/25/2009). Animals were maintained and processed in accordance with the NIH Guide for the Care and Use of Laboratory Animals. Upon arrival, rats were individually housed in double-sized, suspended stainless-steel cages located in an environmentally controlled, pathogen-free facility with a 12-h light/12-h dark cycle. Rats were allowed to acclimate at the animal facility for one week and fed a Purina Rat Chow #5012 (Ralston-Purina, St. Louis, MO, USA). Then, OVX rats were randomly assigned to four dietary groups in a 2 × 2 factorial design: NF (10% fat as energy) or HF (45% fat as energy) and low Ca (LC, 1 g Ca/4057 kcals) or normal Ca (NC, 6 g/4057 kcals). Sham rats were fed a NFNC diet as a control for the OVX procedure. Rats were fed the respective experimental diet for 4 months. The experimental diets (Table 1) were designed and prepared based on the AIN-93M mature rodent diet (NFNC) by Research Diet, Inc. (New Brunswick, NJ, USA). Rats had free access to deionized water throughout the study. Food intake was measured on 4 consecutive days before sacrifice.

### 2.2. Sample Collection and Preparation

Rats were euthanized with intraperitoneal injection of a cocktail at 58 and 8.4 mg/kg body weight for ketamine and xylazine (Animal Health Co. and Phoenix Scientific, St. Joseph, MO, USA), respectively. To minimize the effect of the timing of euthanasia on endpoint measurements, animals were sacrificed between 9:00 a.m. and 11:00 a.m. with a similar number from each group on each day for two consecutive days.

Blood samples were collected via cardiac puncture and centrifuged at 1500× *g* for 20 min at 4 °C. The serum was collected and stored at −80 °C until analyzed. The right tibia of each animal was collected and stored in 70% ethanol before being submitted for structural evaluation, and the femur was collected for dry weight and bone mineral analysis.

### 2.3. Body Composition

Fat mass and lean mass were measured with an EchoMRI-500 whole body composition analyzer (Echo Medical Systems, LLC, Houston, TX, USA) according to the manufacturer’s instruction 3 days before rats were euthanized. The analyzer measures fat mass, lean mass, and free water in scanned specimens using quantitative magnetic resonance theory, and this method has been validated in rats [13]. A clear plastic container of canola oil with a similar weight to the experimental rat was placed in the machine and tested as a fat standard before measuring body composition. Individual live rats were placed in a clear holder. The holder was then placed into a tubular space in the EchoMRI system and activated through a software program. Fat is expressed as equivalent weight in canola oil. Lean is the muscle tissue mass equivalent of all body parts containing water, excluding fat, bone minerals, hair, claws, etc. Free water comes mostly from the bladder.

### 2.4. Bone Structural and Mechanical Evaluation with Micro Computed Tomography (µCT)

The proximal and mid-diaphysis (between the top of the femur head and the bottom of the lateral and medial condyles) of the tibia were scanned and bone structural properties was evaluated using a Scanco µCT scanner (µCT-40; Scanco Medical AG, Bassersdorf, Switzerland) at 16 µm isotropic voxel size with X-ray source power of 55 kV and 145 µA and integration time of 300 milliseconds. The grey-scale images were processed using a low-pass Gaussian filter (sigma = 0.8, support = 1) to remove noise, and a fixed threshold of 275 was used to extract the mineralized bone from the soft tissue and marrow phase. The region of interest (ROI) consisted of 100 slices starting from about 1 mm distal to growth plate for trabecular bone evaluation or 100 slices at the tibial mid-diaphysis for assessment of cortical indexes. For trabecular bone, the following 3D parameters in the defined ROI were reported: bone volume (BV, mm^3^), tissue (cortical and marrow) volume (TV, mm^3^), relative bone volume over total volume (BV/TV, %), trabecular number (Tb.N, 1/mm), trabecular thickness (Tb.Th, mm), trabecular separation (Tb.Sp, mm), connectivity density (Conn.Dn, 1/mm^3^), structure model index (SMI, ranges from 0 to 3 with 0 = plate-like and 3 = rod-like), and BMD (g hydroxyapatite/cm^3^). BMD is the average density of the segmented fraction of the ROI, not including the marrow cavity. For cortical bone evaluation, TV, BV/TV, bone surface (mm^2^), and cortical thickness (Ct.Th, mm) were reported. A calcium hydroxyapatite (HA) phantom (from 0 to 1000 mg/cm^3^) is used for instrument calibration. The recommended guidelines for µCT scanning and nomenclature were followed [14].

### 2.5. Femur Dry Weight and Mineral Analysis

The femurs were dried overnight at 95 °C, weighed, and ashed at 180 °C with 15.8 N HNO_3_ (“Ultrex” grade, J.T. Baker Chemical Co., Phillipsburg, NJ, USA). Ashed bone samples were diluted with 0.1 N HC1 (“Ultrex” grade, J.T. Baker Chemical Co., Phillipsburg, NJ, USA) and subsequently analyzed using inductively coupled argon plasma atomic emission spectroscopy (ICP/6500, Perkin-Elmer, Norwalk, CT, USA). Standard reference material #1515, Apple Leaves (National Institute of Standards and Technology, Gaithersburg, MD, USA), was used for quality control.

### 2.6. Measurements of Serum Biochemical Markers

The following bone-related serum cytokines were measured using commercial rat enzyme-linked immunosorbent assay (ELISA) kits according to the manufacturers’ instructions: leptin (ALPCO Diagnostics, Windham, NH, USA), tartrate-resistant acid phosphatase (TRAP5b, Immunodiagnostic System, Fountain Hill, AZ, USA), and osteocalcin (Biomedical Technologies Inc, Stoughton, MA, USA). To minimize variation, the same cytokine for all samples was measured in one batch preparation on the same day.

### 2.7. Statistical Analyses

Data are expressed as the mean ± SD. The main effects of dietary fat and Ca of OVX groups and their interactions were analyzed using the 2-factor ANOVA (JMP, version 17.0.0; SAS Institute, Inc., Cary, NC, USA). Tukey–Kramer post hoc contrasts were performed to compare group means when the dietary fat by dietary Ca interaction was significant. An a priori contrast was written to test whether the mean of the four OVX groups differed from the Sham. For all analyses, *p* < 0.05 was considered statistically significant.

## 3. Results

**Figure 1:**
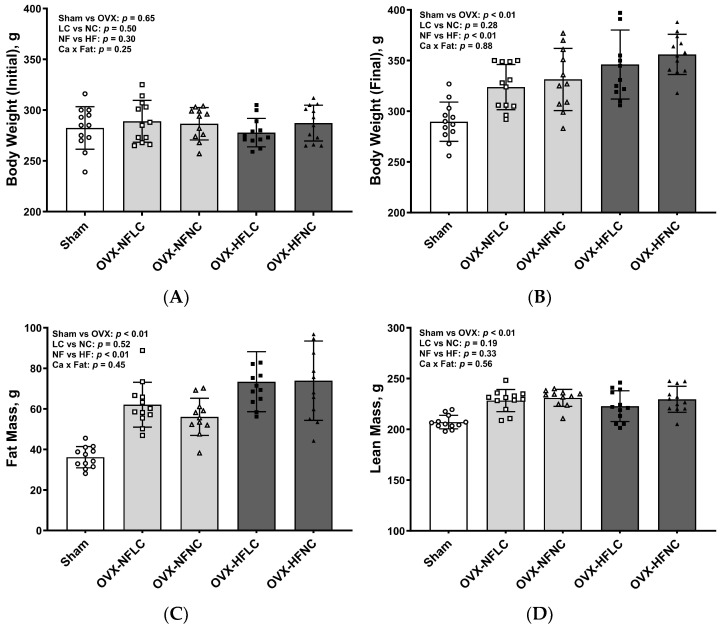
Initial body weight (**A**), final body weight (**B**), fat mass (**C**), and lean mass (**D**) of Sham or OVX rats fed either a purified normal-fat (10% energy as fat) or a high-fat (45% energy as fat) diet containing 1 or 6 g Ca/4057 kcal for 4 months. Data are mean ± SD (*n* = 12 for each group except *n* = 11 for NFNC). The main effects of dietary fat and dietary Ca of OVX groups and their interaction were analyzed using 2-factor ANOVA (JMP, version 17.0.0; SAS Institute, Inc., Cary, NC, USA). Tukey–Kramer post hoc contrasts were performed to compare group means if the dietary fat by dietary Ca interaction was significant. An a priori contrast was written to test whether the mean of the four OVX groups differed from the Sham. For all analyses, *p* < 0.05 was considered statistically significant. HFLC, high-fat low-Ca diet (45% energy as fat, 1 g Ca/4057 kcal); HFLC, high-fat normal-Ca diet (45% energy as fat, 6 g Ca/4057 kcal); NFNC, normal-fat normal-Ca diet (10% energy as fat, 6 g Ca/4057 kcal); NFLC, normal-fat low-calcium diet (10% energy as fat, 1 g Ca/4057 kcal); OVX, ovariectomy (ovariectomized); Sham, Sham-operated.

There were no differences in initial body weight between LC and NC or between NF and HF groups (Figure 1A). Compared to the NF diet, consuming the HF diet increased body weight (Figure 1B, main effect fat: *p* < 0.01) and fat mass (Figure 1C, *p* < 0.01) but not lean mass (Figure 1D, *p* = 0.33). Dietary Ca content did not affect either body weight (main effect Ca: *p* = 0.28), fat mass (*p* = 0.52), or lean mass (*p* = 0.19). There were no significant interactions between dietary Ca and dietary fat for body weight, fat, or lean mass. Pooled OVX groups had greater body weight (*p* < 0.01), fat mass (*p* < 0.01), and lean mass (*p* < 0.01) at the end of the study than those of sham rats.

**Figure 2:**
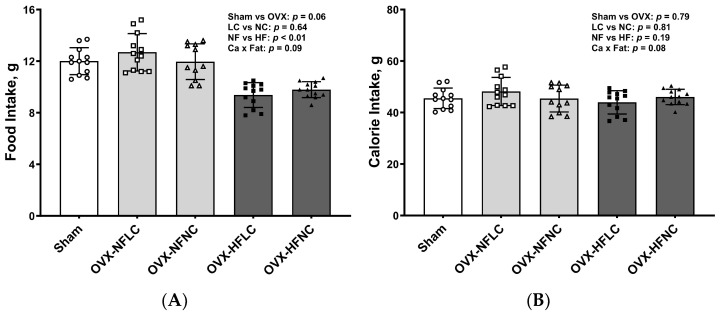
Food intake (**A**) and calorie intake (**B**) of Sham or OVX rats fed either a purified normal-fat (10% energy as fat) or a high-fat (45% energy as fat) diet containing 1 or 6 g Ca/4057 kcal for 4 months. Data are mean ± SD (*n* = 12 for each group except *n* = 11 for NFNC). The main effects of dietary fat and dietary Ca of OVX groups and their interaction were analyzed using the 2-factor ANOVA (JMP, version 17.0.0; SAS Institute, Inc., Cary, NC, USA). Tukey–Kramer post hoc contrasts were performed to compare group means if the dietary fat by dietary Ca interaction was significant. An a priori contrast was written to test whether the mean of the four OVX groups differed from the Sham. For all analyses, *p* < 0.05 was considered statistically significant. HFLC, high-fat low-Ca diet (45% energy as fat, 1 g Ca/4057 kcal); HFLC, high-fat normal-Ca diet (45% energy as fat, 6 g Ca/4057 kcal); NFNC, normal-fat normal-Ca diet (10% energy as fat, 6 g Ca/4057 kcal); NFLC, normal-fat low-calcium diet (10% energy as fat, 1 g Ca/4057 kcal); OVX, ovariectomy (ovariectomized); Sham, Sham-operated.

Food intake and energy intake did not differ between the LC and NC groups (Figure 2A,B). Food intake by animals fed the HF diet was less than those fed the NF diet (main effect Fat: *p* < 0.01). However, total energy intake was the same between the NF and the HF groups (Fat: *p* = 0.19) due to the greater energy content of the HF diet (3.85–3.89 vs. 4.73–4.80 kcal/g for the NF and HF diet as calculated, respectively). Food intake (*p* = 0.06) and energy intake (*p* = 0.79) did not differ between Sham and pooled OVX groups.

**Figure 3:**
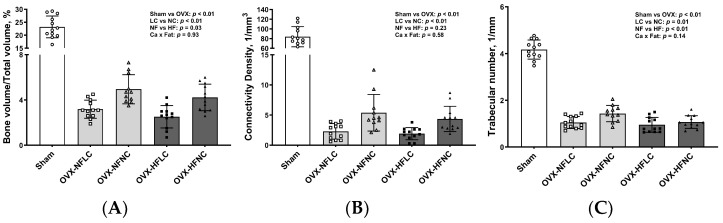

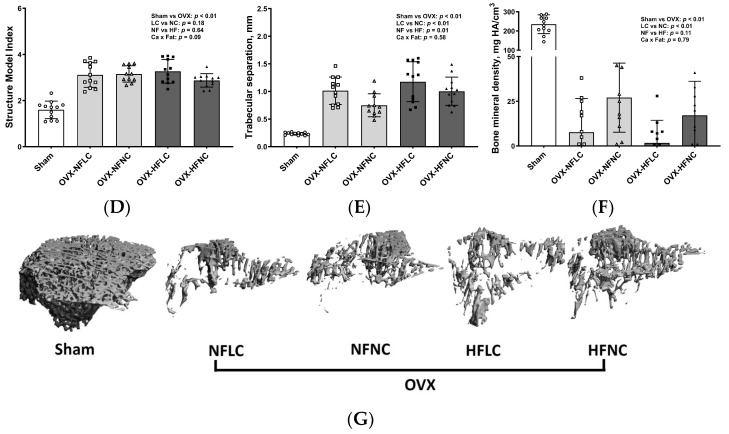
Proximal tibia trabecular BV/TV (**A**), Conn.Dn (**B**), Tb.N (**C**), SMI (**D**), Tb.Sp (**E**), BMD (**F**), and representative images (**G**) of Sham or OVX rats fed either a purified normal-fat (10% energy as fat) or a high-fat (45% energy as fat) diet containing 1 or 6 g Ca/4057 kcal for 4 months. Data are mean ± SD (*n* = 12 for each group except *n* = 11 for NFNC). The region of interest consisted of 100 slices scanned at 16 µm isotropic voxel size starting from about 1 mm distal to the growth plate for trabecular bone evaluation. The main effects of dietary fat and dietary Ca of OVX groups and their interaction were analyzed using the 2-factor ANOVA (JMP, version 17.0.0; SAS Institute, Inc., Cary, NC, USA). Tukey–Kramer post hoc contrasts were performed to compare group means if the dietary fat using dietary Ca interaction was significant. An a priori contrast was written to test whether the mean of the four OVX groups differed from the Sham. For all analyses, *p* < 0.05 was considered statistically significant. BMD, bone mineral density; BV/TV, bone volume/Total volume; Conn.Dn, connectivity density; HFLC, high-fat low-Ca diet (45% energy as fat, 1 g Ca/4057 kcal); HFLC, high-fat normal-Ca diet (45% energy as fat, 6 g Ca/4057 kcal); NFNC, normal-fat normal-Ca diet (10% energy as fat, 6 g Ca/4057 kcal); NFLC, normal-fat low-calcium diet (10% energy as fat, 1 g Ca/4057 kcal); OVX, ovariectomy (ovariectomized); Sham, Sham-operated; SMI, structure model index; Tb.N, trabecular number, Tb.Sp, trabecular separation; Tb.Th, trabecular thickness.

**Figure 4:**
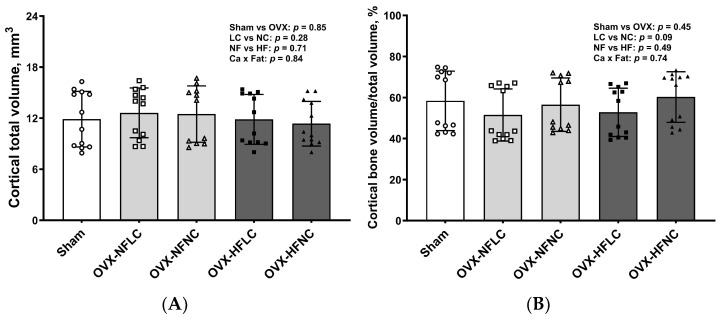

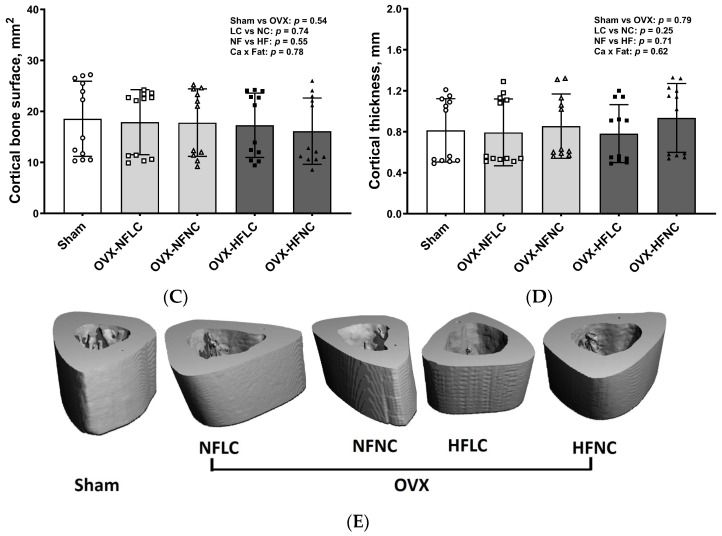
Cortical total volume (**A**), Bone volume/Total volume (**B**), bone surface (**C**), cortical thickness (**D**), and representative images (**E**) of tibial mid-diaphysis of Sham or OVX rats fed either a purified normal-fat (10% energy as fat) or a high-fat (45% energy as fat) diet containing 1 or 6 g Ca/4057 kcal for 4 months. Data are mean ± SD (*n* = 12 for each group except *n* = 11 for NFNC). The region of interest consisted of 100 slices scanned at 16 µm isotropic voxel size at the tibial mid-diaphysis for trabecular bone evaluation. The main effects of dietary fat and dietary Ca of OVX groups and their interaction were analyzed using the 2-factor ANOVA (JMP, version 17.0.0; SAS Institute, Inc., Cary, NC, USA). Tukey–Kramer post hoc contrasts were performed to compare group means if the dietary fat by dietary Ca interaction was significant. An a priori contrast was written to test whether the mean of the four OVX groups differed from the Sham. For all analyses, *p* < 0.05 was considered statistically significant. HFLC, high-fat low-Ca diet (45% energy as fat, 1 g Ca/4057 kcal); HFLC, high-fat normal-Ca diet (45% energy as fat, 6 g Ca/4057 kcal); NFNC, normal-fat normal-Ca diet (10% energy as fat, 6 g Ca/4057 kcal); NFLC, normal-fat low-calcium diet (10% energy as fat, 1 g Ca/4057 kcal); OVX, ovariectomy (ovariectomized); Sham, Sham-operated.

Changes of trabecular bone at the proximal tibia (Figure 3) and cortical bone (Figure 4) at the mid-diaphysis of the tibia in response to dietary Ca and fat were evaluated using a non-destructive μCT instrument. Compared to NC, LC resulted in lower BV/TV (main effect Ca: *p* < 0.01), Conn.Dn (*p* < 0.01), Tb.N (*p* = 0.01), BMD (*p* < 0.01), and greater Tb.Sp at the proximal tibia. Compared to rats on the NF diet, animals fed HF had lower BV/TV (main effect fat: *p* = 0.03) and Tb.N (*p* < 0.01) with greater Tb.Sp (*p* = 0.01). As expected, OVX significantly negatively affected the trabecular bone structural parameters of the tibia. For example, trabecular bone volume and trabecular number decreased by 84% (*p* < 0.01) and 74% (*p* < 0.01), respectively, while trabecular separation increased by 330% (*p* < 0.01). Compared to Sham, OVX rats also had lower tibial Conn.Dn (*p* < 0.01) with greater SMI (*p* < 0.01). Cortical bone parameters were not changed by either dietary Ca, fat, or OVX procedure.

The LC resulted in lower femur weight (Table 2, main effect Ca: *p* < 0.01) and Ca content in the femur (*p* < 0.01). The HF diet increased femur Ca (main effect fat: *p* < 0.01), Ca/P ratio (*p* < 0.01), Cu (*p* = 0.03), and Zn content (*p* < 0.01). Femur Mg, Fe, and P content were not affected by dietary Ca or fat. OVX animals had lower femur weight/body weight (*p* < 0.01), Cu (*p* < 0.01), and Mn (*p* = 0.03) content in the femur than those of the Sham and OVX procedure did not affect other mineral content in the femur.

**Figure 5:**
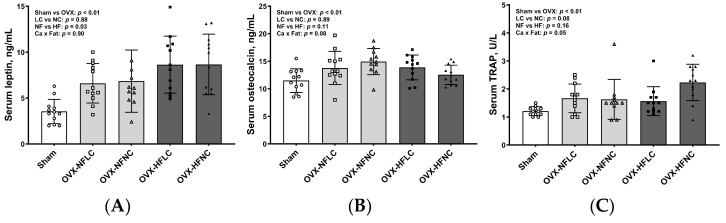
Serum concentrations of leptin (**A**), osteocalcin (**B**), and tartrate-resistant acid phosphatase (**C**) of Sham or OVX rats fed either a purified normal-fat (10% energy as fat) or a high-fat (45% energy as fat) diet containing 1 or 6 g Ca/4057 kcal for 4 months. Data are mean ± SD (*n* = 12 for each group except *n* = 11 for NFNC). The main effects of dietary fat and dietary Ca of OVX groups and their interaction were analyzed using the 2-factor ANOVA (JMP, version 17.0.0; SAS Institute, Inc.). Tukey–Kramer post hoc contrasts were performed to compare group means if the dietary fat by dietary Ca interaction was significant. An a priori contrast was written to test whether the mean of the four OVX groups differed from the Sham. For all analyses, *p* < 0.05 was considered statistically significant. HFLC, high-fat low-Ca diet (45% energy as fat, 1 g Ca/4057 kcal); HFLC, high-fat normal-Ca diet (45% energy as fat, 6 g Ca/4057 kcal); NFNC, normal-fat normal-Ca diet (10% energy as fat, 6 g Ca/4057 kcal); NFLC, normal-fat low-calcium diet (10% energy as fat, 1 g Ca/4057 kcal); OVX, ovariectomy (ovariectomized); Sham, Sham-operated.

HF diet increased serum concentrations of leptin (Figure 5, *p* = 0.03), but not osteocalcin and TRAP. There were no differences in serum cytokine concentrations between LC and NC groups. Compared to Sham, OVX animals had greater serum concentrations of leptin (*p* < 0.01), osteocalcin (*p* < 0.01), and TRAP (*p* < 0.01).

## 4. Discussion

In this study, we examined whether Ca deficiency compromises bone health and modulates fat mass in HF diet-fed obese OVX rats. The results of the study demonstrate that ovariectomy-induced trabecular bone loss. Either a high-fat diet or Ca deficiency resulted in further tibial trabecular bone structure deterioration in OVX rats. Ca deficiency did not affect either body weight or fat mass. There were no interactions between the high-fat diet and dietary Ca content on bone structural parameters, indicating that the high-fat diet and Ca have independent rather synergistic negative effects on bone in OVX rats.

We used ovariectomized rats as a model because bone loss in women with estrogen deficiency following menopause is by far the most common cause of age-related bone loss, accounting for 80% of individuals with osteoporosis in humans [15]. As expected, the proximal tibial trabecular bone structure was severely compromised by the OVX procedure. We also found that ovariectomy resulted in greater adiposity compared to Sham rats, a finding that is consistent with other reports in humans and animals [16,17,18]. Previously, we found that OVX mice had greater body weight, although body composition was not measured in that study, starting about 3 weeks after ovariectomy [19]. Consistent with the increase in fat mass in this study, we found that OVX rats had greater serum concentration of leptin, a cytokine secreted by adipocytes, than Sham. Serum concentration of estradiol or uterine weight could have been measured as positive indicators of OVX. However, changes in bone structure, fat mass, and serum leptin concentrations combined with an increase in bone resorption marker, TRAP, indicate the success of the ovariectomy procedure.

The normal calcium and normal fat diet was formulated based on the AIN-93M mature rodent diet. Rodents consume the same amount of energy regardless of dietary fat content. Therefore, the diets used in this study were formulated based on total energy content to ensure animals had the same intake of other essential nutrients, such as vitamins and minerals that could affect bone metabolism in addition to dietary Ca, which was manipulated in this experiment to study its effects on bone and body composition. As expected, rats fed the HF diet had lower food intake but consumed the same amount of energy compared to those fed the NF diet, which is consistent with the experimental design and the previous studies from our lab and other groups [20,21]. Therefore, any observed differences in endpoint measurements among treatments in this study should be the consequences of either dietary fat or Ca. Hence, the diet formulation based on the energy content should be considered as one of the study strengths.

The impact of obesity on bone has been investigated in animal models and human studies [5,6]. As expected, rats fed the HF diet had 25% greater fat mass compared to those fed the NF diet but had 17% lower BV/TV and 24% greater Tb.Sp at the proximal tibia, a finding similar to previous reports with rats or mice with normal ovary function [8,22,23] or in OVX mice [19]. Research conducted in our lab and by others demonstrated that obesity affects bone, most likely through increased osteoclast activity (bone resorption) and/or decreased osteoblast activity (bone formation), as well as elevated adipose tissue inflammation [8,22,23,24].

Data showing an inverse association between dietary Ca and obesity in humans were first reported by Zemel et al. [25] and then by others [11,26,27]. It is hypothesized that low Ca intake would increase adipocyte intracellular Ca^2+^ concentration and that greater intracellular Ca^2+^ concentration stimulates lipogenesis while inhibiting lipolysis [11]. However, the results from our study showed that Ca deficiency did not affect food intake, body weight, or fat mass in OVX rats, suggesting that the effect of Ca on bone is not likely through modulating adiposity. It is possible that the increase in fat mass with the OVX procedure could diminish the impact of Ca deficiency on fat mass or that older animals (10-month-old at the time of sacrifice in this study) are less responsive to Ca deficiency with regard to the impact on adiposity, as compared to young growing animals in another study [11]. The effect of Ca intake on adiposity remains controversial. The inverse [11,25,26,27], or lack of relationships, has both been reported in animal and human studies [28,29,30,31]. For example, Shapses et al. conducted three 25-week randomized, double-blind, placebo-controlled trials and reported no differences in either body weight or fat mass in premenopausal and postmenopausal women supplemented with 1000 mg Ca/d compared to those without Ca supplementation [30]. Zhang and Tordoff (2004) reported that energy intake, body weight, or carcass fat content were not affected by Ca intake ranging from 2, 6, and 18 g/kg diet in female Sprague–Dawley rats fed either normal- or high-fat diet [31]. Apparently, more studies are needed because the effects of Ca intake on obesity, if any, may be influenced by many factors, such as animal species, duration of supplementation or deficiency, or other physiological or environmental factors [9,10].

In this study, we found that either Ca deficiency or a HF diet did not affect mid-shaft cortical parameters of the tibia even though Ca deficiency resulted in lower femur weight and Ca content, and the HF diet even increased the femur Ca content. The finding that cortical bone was not affected by the HF diet is consistent with our previous study with rats that were fed a HF diet [22]. However, it is quite unexpected that the cortical procedure did not affect the OVX parameters, considering trabecular bone mass decreased by over 80% in OVX rats compared to Sham rats. One possible explanation is that, in general, cortical bone is less responsive than trabecular bone to changes in diets, physical or physiological factors, or even drug treatments because cortical bone has a smaller surface exposed to bone marrow and that the blood flow and turnover is lower than in trabecular bone, depending on the location [32,33]. For example, in humans, bone loss for the first 10 years after menopause is more rapid in the trabecular bone than in the cortical bone [34]. Therefore, an increase in study duration might be needed to achieve detectable changes in cortical bone.

The use of micro-CT in determining changes in trabecular and cortical bone structure among dietary treatments is another strength of the study. While bone mineral analysis is useful in providing overall distribution and/or changes of individual minerals in bone, it does not index bone structure or differentiate bone compartments, i.e., trabecular and cortical bone, and, therefore, cannot predict bone strength. Unexpectedly, we found that the HF diet increased the Ca, Cu, and Zn content of the femur. These changes may reflect that HF affects various bone compartments differently and requires further investigation. Although cortical bone makes up approximately 80% of total skeletal tissue mass, trabecular bone structure is a more important determinant of bone strength [34]. For example, intermittent parathyroid hormone treatment increased trabecular bone mass and biomechanical bone strength despite a decrease in cortical bone mass [35]. The limitation of the study was that bone strength, which can be determined by three-point bending or a finite element analysis, was not measured.

## 5. Conclusions

In summary, our data demonstrate that obesity induced by a high-fat diet is detrimental to bone structure. Ca deficiency did not affect body weight or fat mass and had an independent negative effect on bone microstructure in ovariectomized rats. The findings suggest that reducing excessive adiposity or maintaining adequate Ca intake is important for bone health animals with impaired ovary function. Whether Ca supplementation above the requirement can prevent or reduce the high-fat diet-induced bone structural deterioration should be explored in future studies.

## Figures and Tables

**Table 1:** Composition of experimental diets ^1^.

Ingredient	NFLC	NFNC	HFLC	HFNC
	g			
Casein, 80 Mesh	200	200	200	200
L-Cystine	3.0	3.0	3.0	3.0
Corn starch	315	315	72.8	72.8
Maltodextrin 10	35	35	100	100
Sucrose	350	350	172.8	172.8
Cellulose, BW200	50	50	50	50
Soybean oil	25.0	25.0	25.0	25.0
Lard	20	20	177.5	177.5
Mineral mix ^2^	10.0	10.0	10.0	10.0
Dicalcium phosphate	3.4	13.0	3.4	13.0
Calcium carbonate	0	5.5	0	5.5
Potassium citrate, H_2_O	8.89	16.5	8.89	16.5
Potassium phosphate	9.6	0	9.6	0
Vitamin mix ^3^	10.0	10.0	11.1	12.5
Choline bitartrate	2.0	2.0	2.2	2.5
Total weight, g	1042	1055	845	858
Energy, kcal/g diet	3.89	3.85	4.80	4.73
% Energy				
Carbohydrate	70	70	35	35
Protein	20	20	22	25
Fat	10	10	45	45
Ca, g/4057 kcal	1.0	6.0	1.0	6.0

^1^ Diets were designed and prepared by Research Diet, Inc., New Brunswick, NJ, USA, and micronutrients were formulated based on the energy content. Quality control steps were conducted during the diet manufacturing process to ensure that the checklists (the list of ingredients in the diet) are made as per the formulation. ^2^ The mineral mix composition was as follows (amount in 10 g): 0.5 g Mg, 0.3 g S, 1.0 g Na, 1.6 g Cl, 6.0 mg Cu, 0.2 mg I, 45.0 mg Fe, 59 mg Mn, 0.2 mg Se, and 29 mg Zn. ^3^ The vitamin mix composition was as follows (amount in 10 g): 4000 IU vitamin A palmitate, 1000 IU cholecalciferol, 50 IU vitamin E acetate, 0.5 mg menadione sodium bisulfite, 0.2 mg biotin, 10 μg cyanocobalamin, 2 mg folic acid, 30 mg nicotinic acid, 16 mg calcium pantothenate, 7 mg pyridoxine-HCl, 6 mg riboflavin, and 6 mg thiamin HCl.

**Table 2:** Femur weight and mineral content from Sham or OVX rats fed either a purified normal-fat (10% energy as fat) or a high-fat (45% energy as fat) diet containing 1 or 6 g Ca/4057 kcal for 4 months ^1^.

	Weight (mg)	Femur Weight/BW (%)	Calcium (mg/g)	Phosphorus (mg/g)	Ca:P	Magnesium (mg/g)	Copper (µg/g)	Iron (µg/g)	Zinc (µg/g)	Manganese (µg/g)
Sham	690 ± 73	0.24 ± 0.01	220 ± 19	89.1 ± 7.4	2.47 ± 0.21	2.14 ± 0.16	1.70 ± 0.48	57.3 ± 11.2	216 ± 15	0.80 ± 0.04
OVX-NFLC	653 ± 35	0.20 ± 0.02	194 ± 21	90.1 ± 4.7	2.16 ± 0.28	2.15 ± 0.10	1.13 ± 0.09	52.2 ± 5.1	219 ± 17	0.76 ± 0.05
OVX-NFNC	682 ± 62	0.21 ± 0.02	210 ± 18	90.4 ± 6.9	2.33 ± 0.20	2.13 ± 0.11	1.14 ± 0.10	48.8 ± 8.8	212.5 ± 11.5	0.74 ± 0.06
OVX-HFLC	657 ± 38	0.19 ± 0.01	221 ± 8	90.8 ± 5.2	2.44 ± 0.11	2.11 ± 0.05	1.17 ± 0.17	54.1 ± 9.8	227.3 ± 9.5	0.74 ± 0.06
OVX-HFNC	699 ± 38	0.20 ± 0.02	238 ± 21	92.2 ± 2.6	2.59 ± 0.24	2.19 ± 0.07	1.26 ± 0.07	56.3 ± 10.4	234.0 ± 10.6	0.78 ± 0.05
Ca	<0.01	0.36	<0.01	0.57	0.02	0.30	0.22	0.82	0.93	0.47
Fat	0.41	0.04	<0.01	0.38	<0.01	0.67	0.04	0.07	<0.01	0.37
Ca × Fat	0.57	0.85	0.93	0.70	0.84	0.06	0.37	0.28	0.09	0.09
Sham vs. OVX	0.28	<0.01	0.63	0.33	0.27	0.87	<0.01	0.16	0.13	0.03

^1^ Values are the mean ± SD. The main effects of dietary fat and Ca of OVX groups and their interactions were analyzed using 2-factor ANOVA (JMP, version 17.0.0; SAS Institute, Inc., NC, USA). Tukey–Kramer post hoc contrasts were not performed to compare group means if the fat x Ca interaction was not significant. To test for differences between Sham and OVX groups in the one-way ANOVA, a pre-planned contrast was written to compare Sham to all levels of OVX. For all analyses, *p* < 0.05 was considered statistically significant. HFLC, high-fat low-Ca diet (45% energy as fat, 1 g Ca/4057 kcal); HFLC, high-fat normal-Ca diet (45% energy as fat, 6 g Ca/4057 kcal); NFNC, normal-fat normal-Ca diet (10% energy as fat, 6 g Ca/4057 kcal); NFLC, normal-fat low-calcium diet (10% energy as fat, 1 g Ca/4057 kcal); OVX, ovariectomy (ovariectomized); Sham, Sham-operated.

## Data Availability

Data are contained within the article.
